# Patient co-payments for women diagnosed with breast cancer in Australia

**DOI:** 10.1007/s00520-019-05037-z

**Published:** 2019-08-21

**Authors:** Nicole Bates, Emily Callander, Daniel Lindsay, Kerrianne Watt

**Affiliations:** 1 College of Public Health, Medical and Veterinary Sciences (CPHMVS), James Cook University (JCU), Townsville, Australia; 2 Australian Institute of Tropical Health and Medicine, JCU, Townsville, Australia; 3 Centre for Applied Health Economics, School of Medicine, Griffith University, Nathan, Australia

**Keywords:** Breast cancer, Patient co-payment, Financial toxicity, Australia

## Abstract

**Purpose:**

Among Australian women, breast cancer is the most commonly diagnosed cancer. The out-of-pocket cost to the patient is substantial. This study estimates the total patient co-payments for Medicare Benefits Schedule (MBS) and Pharmaceutical Benefits Scheme (PBS) for women diagnosed with breast cancer and determined the distribution of these costs by Indigenous status, remoteness, and socioeconomic status.

**Methods:**

Data on women diagnosed with breast cancer in Queensland between 01 July 2011 and 30 June 2012 were obtained from the Queensland Cancer Registry and linked with hospital and Emergency Department Admissions, and MBS and PBS records for the 3 years post-diagnosis. The data were then weighted to be representative of the Australian population. The co-payment charged for MBS services and PBS prescriptions was summed. We modelled the mean co-payment per patient during each 6-month time period for MBS services and PBS prescriptions.

**Results:**

A total of 3079 women were diagnosed with breast cancer in Queensland during the 12-month study period, representing 15,335 Australian women after weighting. In the first 3 years post-diagnosis, the median co-payment for MBS services was AU$ 748 (IQR, AU$87–2121; maximum AU$32,249), and for PBS prescriptions was AU$ 835 (IQR, AU$480–1289; maximum AU$5390). There were significant differences in the co-payments for MBS services and PBS prescriptions by Indigenous status and socioeconomic disadvantage, but none for remoteness.

**Conclusions:**

Women incur high patient co-payments in the first 3 years post-diagnosis. These costs vary greatly by patient. Potential costs should be discussed with women throughout their treatment, to allow women greater choice in the most appropriate care for their situation.

## Introduction

In 2018, it was anticipated that an estimated 18,087 women would be diagnosed with breast cancer, which is the most commonly diagnosed cancer in women within Australia [1]. Most recent estimates suggest that 5-year survival for women diagnosed with breast cancer in Australia is 90% [1]. However, improvements in treatment and survival come at a cost to both the healthcare system and the patient. Recent Australian studies have highlighted that women diagnosed with breast cancer will face significant out-of-pocket (OOP) costs [2–4]. These high OOP costs may result in people diagnosed with cancer delaying or forgoing healthcare [5–7]. This financial burden placed on individuals and their families due to a cancer diagnosis is known as ‘financial toxicity’ [8].

Australia has a universal healthcare system, Medicare, which has three parts: hospital, medical, and prescription pharmaceutical. Individuals receive free treatment at public hospitals and free or subsidized medical services outside of public hospitals. The Medicare Benefits Schedule (MBS) includes medical services such as attendances by medical doctors, tests and scans, most procedures performed by doctors, optometrists, and some allied health services. For items listed on the MBS, Medicare pays a rebate (proportion of the fee) to the service provider. If the fee charged is equal to the rebate, the patient incurs no co-payment (the service is ‘bulk-billed’); however, if the fee charged by the service provider is greater than the rebate, the patient will be charged an OOP co-payment [9]. Medical service providers in Australia may set their own fees, resulting in unregulated OOP fees for patient [10].

The Pharmaceutical Benefits Scheme (PBS) is a list of approved prescription pharmaceuticals, for which the patient is charged a co-payment, and the Australian Government funds the remainder of the fee [9]. There are also a number of policies in place to protect individuals and family groups from spending a high amount on patient co-payments during the year. Individuals and families with a concession card or health care card may be eligible to obtain medications and health services at a lower cost. In addition to this, the respective Medicare and PBS Safety Nets have a number of thresholds which depend on the individual or family group circumstances, such as concessional cardholders. Once an individual or family group reach the threshold, they will have a higher proportion of their service/prescription subsidized for the remainder of the year [9]. In 2010, the Closing the Gap (CTG) PBS prescriptions were introduced, which allowed Indigenous Australians to have access to PBS medicines at a lower cost or for free [11].

There is growing concern regarding the high OOP expenditure associated with a breast cancer diagnosis. A recent report commissioned by the Breast Cancer Network Australia indicated that the median OOP for women was $4809 in the first 5 years post-diagnosis (interquartile range (IQR), $1510 and $17,200) [4]. Although similar OOP costs for women living in urban and non-urban areas were reported, women living in non-urban areas were found to access fewer services. Women living in non-urban areas were also reported to spend more on accommodation costs compared with women from urban areas [4]. In a recent Queensland study using administrative data to estimate the OOP costs of major cancers, the median co-payment for women diagnosed with breast cancer was $4192 (IQR, 1165–7459) during the first 2 years following diagnosis [3]. However, the sample was relatively small (84 women diagnosed with breast cancer), and the study did not compare costs by sub-populations such as Indigenous status, remoteness, or socioeconomic status. A longitudinal study of 287 Queensland women diagnosed with breast cancer found that the greatest total costs (direct and indirect) were during the first 6 months post-diagnosis, followed by a gradual decline over the next 18 months [2]. Costs were higher for women diagnosed with positive lymph nodes and for younger women (≤ 50 years) [2]. In a study of people diagnosed with cancer and being treated at The Townsville Hospital (Queensland), travel expenses accounted for the greatest OOP costs (71%), followed by medical services (10%) and medications (9%) [12]. Costs were highest for people living further away from the hospital and for people receiving radiotherapy [12]. The two latter studies relied on self-reported costs, which have some limitations associated with recall. The use of linked administrative data may overcome this shortcoming.

A linked administrative data model (CancerCostMod) was used in the present study to estimate the patient co-payments for women diagnosed with breast cancer. For this study, we adopted an individual perspective to (1) estimate the total patient co-payments for MBS services and PBS prescriptions for women diagnosed with breast cancer during the first 3 years following diagnosis and (2) determine the distribution of these co-payment costs by Indigenous status, remoteness, and socioeconomic status.

## Methods

### Study population

The methodology for ‘CancerCostMod’ has been previously described [13]. Briefly, this dataset comprises all cancer diagnoses (excluding non-melanoma skin cancer) in the Queensland Cancer Registry (QCR) between 01 July 2011 and 30 June 2012 (*N* = 25,553 patients), which were then linked with data on hospital admissions (Queensland Health Admitted Patient Data Collection (QHAPDC)) ED presentations (Emergency Department Information Systems (EDIS)), MBS, and PBS from 01 July 2011 and 30 June 2015. The Queensland Health Statistical Services Branch completed the linkage of QCR, QHAPDC, and EDIS, and then the Australian Institute of Health and Welfare (AIHW) linked this dataset to MBS and PBS. The base population was weighted by the authors to the Australian population to be representative of the Australian population. The authors used a programmed SAS macro, GREGWT (weighted *N* = 123,900) [13]. The 2012 Australian Cancer Database was used as the benchmark for the weighting [14]. For this study, we extracted from ‘CancerCostMod’ records of female breast cancer (ICD-O C50) in those aged 18 years or greater at the time of diagnosis.

### Sociodemographic characteristics

Sociodemographic variables obtained in the QCR dataset at the time of diagnosis were age, sex, Indigenous status, and residential postcode. Postcode was mapped to the Index of Relative Socio-Economic Disadvantage (IRSD) and collapsed into quintiles (Q1 = most disadvantaged, and Q5 = least disadvantaged). The IRSD is a summary of the economic and social conditions of an area and is a measure of relative socioeconomic disadvantage [15]. Postcode was also mapped to the Australian Statistical Geography Standard [16] to obtain a measure of remoteness: metropolitan, regional (inner and outer), and remote (remote and very remote). The original QCR dataset had 151 records with missing postcodes were unable to be mapped to IRSD or remoteness. Indigenous status was recorded for 87% of the sample obtained from the QCR. The authors imputed records with missing Indigenous status. Briefly, records of patients with missing Indigenous status who lived in a local government area where ≥ 75% of the population were Indigenous Australian were assigned to be ‘Indigenous.’ We then used multiple imputation to impute the remaining records with missing Indigenous status. These methods have been described in more detail previously [13].

### Breast cancer staging

The stage at diagnosis is not routinely collected by jurisdictional cancer registries in Australia. As such, we categorized stages into ‘early’ (tumour size ≤ 20 mm with no evidence of lymph node involvement), ‘advanced’ (tumour size > 20 mm, or if any lymph node involvement regardless of size, or if there was metastatic disease), and ‘unknown’ (tumour size or lymph node involvement was unknown) using similar methods published in previous Queensland studies [17, 18].

### Assigning patient co-payments to MBS services and PBS prescriptions

The MBS and PBS datasets used in developing CancerCostMod included information on the date of service/prescription, patient postcode, provider postcode, item code, full charge, Government rebate, and patient co-payment. The patient co-payment was summed monthly for MBS services and PBS prescriptions from the date of diagnosis (time = 0) for 36 months following diagnosis. If an individual died during the first 3 years following diagnosis, no costs were recorded for subsequent months following death. All co-payments were adjusted to the 2016–2017 financial year using the Reserve Bank of Australia inflation calculator [19]. All costs are reported in Australian dollars (AUD).

The MBS and PBS datasets include all MBS services and PBS prescriptions, which includes oncology and non-oncology medical services and prescriptions. This study excluded any costs associated with treatment that was not covered by Medicare, such as some medical services, over-the-counter, or private prescriptions. Other OOP costs such as private health insurance, hospital excess or charges, travel, accommodation, food, or indirect costs due to changes in labour force participation for the patient (and their caregiver/s) were also excluded. Patient comorbidities were also excluded from the dataset and, therefore, not adjusted for in the analysis.

### Statistical analysis

Descriptive analyses were conducted to determine the characteristics of women diagnosed with breast cancer. To describe the total co-payment costs for this sample, we aggregated the co-payments for MBS and PBS separately into 6-month time periods from the date of diagnosis (*t* = 0) to 36 months post-diagnosis. We report the total and average patient co-payment separately for MBS services and PBS prescriptions during each of the time periods analysed (limited to those who accessed at least one health event).

Finally, we modelled the mean patient co-payment during each 6-month time period using generalized linear models, using a negative binomial regression, and a log link function. There were six separate models (one for each 6-month period) for MBS co-payments and 6 separate models for PBS co-payments. Covariates included in these analyses were Indigenous status (reference = non-Indigenous women), age group (reference = 18–44 years), remoteness (reference = metropolitan), socioeconomic disadvantage (reference = IRSD Q5 (least disadvantaged)), breast cancer stage (reference = early), number of medical services accessed during the period analysed, and death during the time period being modelled. These are variables that may have influenced treatment and therefore costs associated with treatment. The models also included the number of months the individual survived as an offset to the model. All analyses were undertaken using SAS V9.4 (SAS Institute Inc., Cary, NC, USA).

Human Research Ethics approval was obtained from the Townsville Hospital and Health Service Human Research Ethics Committee (HREC) (HREC/16/QTHS/11), AIHW HREC (EO2017/1/343), and James Cook University HREC (H6678). Permission to waive consent was approved from Queensland Health under the Public Health Act 2005. No identifiable information was provided to the authors.

## Results

Between 1 July 2011 and 30 June 2012, 3079 women were diagnosed with breast cancer in Queensland. This represents 15,335 Australian women once weighted. Demographic characteristics at diagnosis are shown in Table 1. The mean age for this cohort was 61 years (SD, 14 years). Demographic characteristics were similar for the weighted and unweighted sample.

**Table 1:** Demographic characteristics at diagnosis of Australian women diagnosed with breast cancer between 1 July 2011 and 30 June 2012 (weighted)

	*N*
*N*	3079
*N* (weighted)	15,335
Age group	
18–44 years (%)	1848 (12.1)
45–64 years (%)	7536 (49.1)
≥ 64 years (%)	5951 (38.8)
12-month mortality	646 (4.2)
Indigenous status	
Indigenous women (%)	248 (1.6)
Non-Indigenous women (%)	15,087 (98.4)
Remoteness*	
Metropolitan (%)	7712 (50.6)
Regional (%)	6359 (41.7)
Remote (%)	1180 (7.7)
Index of Relative Socio-Economic Disadvantage*	
Quintile 1 (most disadvantaged) (%)	1095 (7.2)
Quintile 2 (%)	767 (5.0)
Quintile 3 (%)	2483 (16.3)
Quintile 4 (%)	6669 (43.7)
Quintile 5 (least disadvantaged) (%)	4236 (27.8)
Breast cancer stage	
Early (%)	6695 (43.6)
Advanced (%)	7174 (46.8)
Unknown (%)	1466 (9.6)

*Those with missing postcode data at diagnosis were excluded (weighted *n* = 85)

During the first 12 months following diagnosis, 646 women passed away. Of this, 44% lived in metropolitan areas, 36% lived in regional areas, and 11% lived in remote areas (please note that due to missing postcode data, this does not add to 100%). Of those who passed away during the first 12 months following diagnosis, 7% lived in the most disadvantaged areas (IRSD Q1), 3% lived in Q2, 16% lived in Q3, 46% lived in Q4, and 20% lived in Q5 (least disadvantaged). Table 2 describes the stages of disease at diagnosis for Australian women diagnosed with breast cancer by Indigenous status, remoteness, and socioeconomic status.

**Table 2:** Stages of disease at diagnosis of Australian women diagnosed with breast cancer between 1 July 2011 and 30 June 2012 (weighted)

	Early	Advanced	Unknown
Indigenous Australian (%)	75 (30)	123 (50)	50 (20)
Non-Indigenous Australian women (%)	6620 (44)	7052 (47)	1415 (9)
Remoteness**			
Major city (%)	3424 (44)	3567 (46)	721 (9)
Regional (%)	2757 (43)	3066 (48)	536 (8)
Remote (%)	514 (44)	537 (46)	129 (11)
Index of Relative Socio-Economic Disadvantage**			
Quintile 1 (most disadvantaged) (%)	439 (40)	557 (51)	99 (9)
Quintile 2 (%)	368 (48)	351 (46)	49 (6)
Quintile 3 (%)	1085 (44)	1188 (48)	210 (9)
Quintile 4 (%)	3018 (45)	3078 (46)	573 (9)
Quintile 5 (least disadvantaged) (%)	1786 (42)	1995 (47)	455 (11)

**Those with missing postcode data at diagnosis were excluded (weighted *n* = 85)

Table 3 describes the number of MBS services and PBS prescriptions accessed by women diagnosed with breast cancer during the first 3 years post-diagnosis. On average, each woman accessed 233 services MBS services (SD, 144) during the first 3 years following a breast cancer diagnosis and an average of 99 PBS prescriptions (SD, 90).

**Table 3:** Number of MBS services and PBS prescriptions for women diagnosed with breast cancer in Australia (weighted)

Time since diagnosis (months)	MBS services	PBS prescriptions
	Mean ± SD	Mean ± SD
0–6	75 ± 45	25 ± 19
7–12	47 ± 39	18 ± 17
13–18	30 ± 27	17 ± 17
19–24	28 ± 31	17 ± 17
25–30	28 ± 34	17 ± 17
31–36	26 ± 31	17 ± 17

A summary of the patient co-payments for MBS services and PBS prescriptions over the first 3 years following diagnosis is reported in Table 4. During the first 3 years post-diagnosis, the average co-payments for MBS services was AU$1440 (SD, $1946). For MBS patient co-payments, the standard deviation was larger than the mean in each of the 6-month periods, indicating a wide dispersion in the average patient co-payment between individuals. This was not observed for PBS prescriptions. During the first 3 years post-diagnosis, the average co-payments for PBS prescriptions was AU$974 (SD, $707).

**Table 4:** Patient co-payments of MBS services and PBS prescriptions for women diagnosed with breast cancer in Australia (weighted)

Time since diagnosis (months)	MBS services				PBS prescriptions			
	Mean ± SD (AU$)	Median (AU$)	Interquartile range (AU$)	Maximum (AU$)	Mean ± SD (AU$)	Median (AU$)	Interquartile range (AU$)	Maximum (AU$)
0–6	649 ± 845	229	1–1121	6620	326 ± 322	205	105–451	2137
7–12	294 ± 605	61	0–253	9404	175 ± 148	141	77–222	1759
13–18	145 ± 314	54	0–164	5901	156 ± 131	121	72–216	1282
19–24	140 ± 390	49	0–149	10,193	153 ± 127	115	73–219	1192
25–30	140 ± 423	54	0–145	9899	145 ± 124	112	61–193	1536
31–36	133 ± 407	44	0–138	10,745	143 ± 118	116	61–192	1203
TOTAL	1440 ± 1946	748	87–2121	32,249	974 ± 707	835	480–1289	5390

**Fig. 1:**
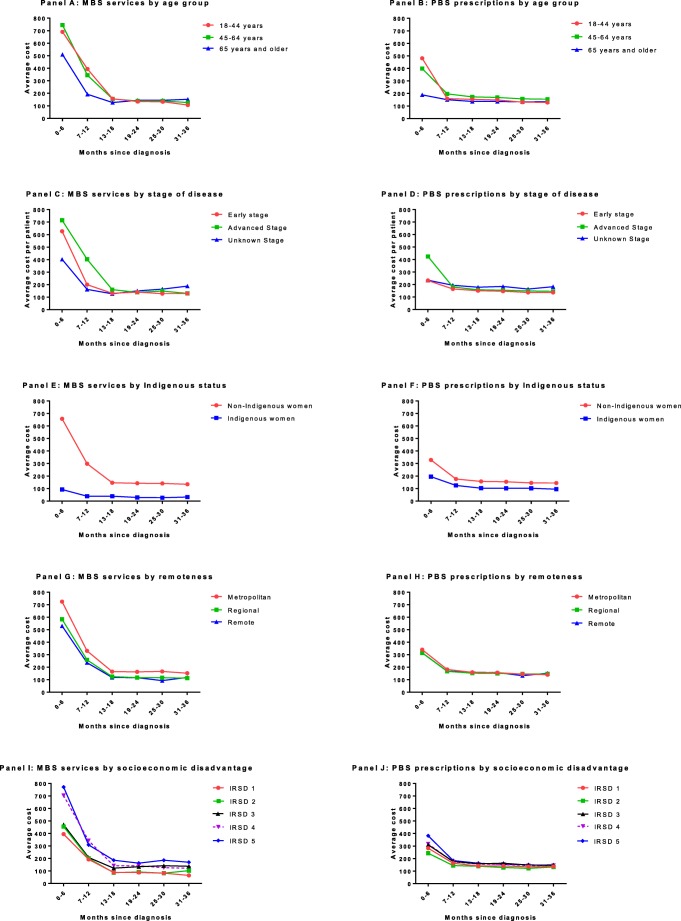
Average patient co-payments for MBS services and PBS prescriptions by age group (**a**, **b**), breast cancer stage (**c**, **d**), Indigenous status (**e**, **f**), remoteness (**g**, **h**), and socioeconomic disadvantage (**i**, **j**). Average costs per patient were calculated for each 6-month period from diagnosis to 3 years. The figures on the left present the unadjusted average patient co-payments for MBS services, and on the right present the unadjusted average patient co-payments for PBS prescriptions by characteristics of interest

The average patient co-payments for MBS services and PBS prescriptions are shown in Fig. 1 by age group (panels a and b), stage of disease (panels c and d), Indigenous status (panels e and f), remoteness (panels g and h), and socioeconomic disadvantage (panels i and j). In most of the panels, the first 6 months following diagnosis accounted for a higher proportion of patient co-payments. There is some variation in the average co-payment for MBS services during the 0–6 and 7–12 months post-diagnosis by age group and stage of disease, but after 12 months, there is little to no variation. There is some variation in the average co-payment for PBS prescriptions over the first 6 months by age group and stage of disease. Indigenous women have lower average patient co-payments for both MBS services and PBS prescriptions across all time periods. There was some variation observed in the average patient co-payment for MBS services when comparing by remoteness and socioeconomic disadvantage. Women living in metropolitan areas appear to have slightly higher co-payments for MBS services throughout the first 3 years compared with women living in regional and remote areas. Women living in the least disadvantaged quintiles (Q4 and Q5) had higher patient co-payments for MBS services compared with those living in quintiles 1–3. There was very little variation in the patient co-payment for PBS prescriptions by remoteness or socioeconomic disadvantage.

Table 5 shows the parameter estimates produced by the six generalized linear models, estimating the mean patient co-payment per patient for each 6-month time period for MBS services, adjusting for Indigenous status, remoteness**,** socioeconomic status, age group at diagnosis, stage of disease at diagnosis, number of MBS services during period analysed, and death during time period being analysed. For MBS services, co-payments were 82% lower in Indigenous women during 0–6 months and 79% lower during the 7–12 months post-diagnosis compared with those in non-Indigenous women. There were no consistent differences between areas of remoteness. Compared with women living in the least disadvantaged area (Q5), women living in Q1, Q2, and Q3 had significantly lower costs for 0–6 months and 7–12 months.

**Table 5:** Parameter estimates of independent variables in generalized linear regression model of the co-payments for MBS services for women diagnosed with breast cancer between 1 July 2011 and 30 June 2012, Australia (weighted data presented)

	0–6 months		7–12 months		13–18 months		19–24 months		25–30 months		31–36 months	
	Ratio	Coefficient (SE)	Ratio	Coefficient (SE)	Ratio	Coefficient (SE)	Ratio	Coefficient (SE)	Ratio	Coefficient (SE)	Ratio	Coefficient (SE)
Intercept		3.2038 (0.1917)***		1.8748 (0.2348)***		2.0679 (0.1605)***		1.9362 (0.1263)***		1.7002 (0.1209)***		1.3809 (0.1364)***
Indigenous women	0.18	− 1.7388 (0.2791)***	0.21	− 1.5685 (0.3202)***	0.99	− 0.0139 (0.2673)	0.82	− 0.1972 (0.2623)	0.65	− 0.4288 (0.2730)	1.06	0.0556 (0.3063)
Regional	1.05	0.0443 (0.0835)	1.02	0.0226 (0.0968)	0.99	− 0.0083 (0.0484)	0.95	− 0.0557 (0.0472)	1.09	0.0896 (0.0454)*	0.99	− 0.0136 (0.0501)
Remote	1.27	0.2370 (0.1440)	1.04	0.0360 (0.1671)	1.01	0.0079 (0.0839)	0.91	− 0.0926 (0.0780)	1.03	0.0264 (0.0781)	0.92	− 0.0816 (0.0853)
IRSD Q1	0.59	− 0.5337 (0.1591)***	0.62	− 0.4783 (0.1860)*	0.74	− 0.3064 (0.0957)**	0.97	− 0.0350 (0.0933)	0.72	− 0.3236 (0.0921)***	0.76	− 0.2694 (0.0985)**
IRSD Q2	0.64	− 0.4427 (0.1797)*	0.49	− 0.7087 (0.2095)***	0.72	− 0.3322 (0.1062)**	0.90	− 0.1021 (0.1021)	0.73	− 0.3095 (0.0978)**	0.83	−0.1923 (0.1070)
IRSD Q3	0.58	− 0.5499 (0.1176)***	0.59	− 0.5270 (0.1348)***	0.86	− 0.1470 (0.0692)	0.94	− 0.0594 (0.0677)	0.78	− 0.2450 (0.0648)***	1.01	0.0079 (0.0714)
IRSD Q4	0.89	− 0.1129 (0.0879)	0.90	− 0.1107 (0.1025)	0.84	− 0.1773 (0.0502)***	0.92	− 0.0799 (0.0478)	0.81	− 0.2084 (0.0468)***	0.88	− 0.1256 (0.0513)*
Age 45–64 years	1.06	0.0604 (0.1090)	1.02	0.0168 (0.1268)	0.92	− 0.0869 (0.0623)	0.98	− 0.0215 (0.0582)	0.95	− 0.0506 (0.0584)	1.10	0.0909 (0.0626)
Age ≥ 65 years	0.71	− 0.3448 (0.1129)**	0.60	− 0.5067 (0.1318)***	0.63	− 0.4545 (0.0662)***	0.73	− 0.3089 (0.0622)***	0.73	− 0.3120 (0.0626)***	0.89	− 0.1214 (0.0677)
Advanced stage	0.73	− 0.3197 (0.0718)***	1.08	0.0801 (0.0876)	1.02	0.0168 (0.0409)	1.03	0.0276 (0.0387)	0.98	− 0.0169 (0.0383)	0.96	− 0.0405 (0.0417)
Unknown stage	0.58	− 0.5402 (0.1365)***	0.63	− 0.4689 (0.1672)**	0.95	− 0.0472 (0.0948)	1.07	0.0655 (0.1003)	1.09	0.0896 (0.1022)	0.81	− 0.2159 (0.1149)
Number of MBS services during period analysed	1.03	0.0260 (0.0011)***	1.03	0.0261 (0.0014)***	1.02	0.0207 (0.0009)***	1.02	0.0188 (0.0007)***	1.02	0.0182 (0.0007)***	1.02	0.0200 (0.0009)***
Death during period analysed	0.62	− 0.4778 (0.1269)***	0.92	− 0.0790 (0.1768)	0.87	− 0.1349 (0.1419)	0.65	− 0.4251 (0.1035)***	0.71	− 0.3416 (0.1000)***	0.72	− 0.3334 (0.1155)**

The ratios presented are relative to the reference group: Indigenous status (reference = non-Indigenous women), age group (reference = 18–44 years), remoteness (reference = metropolitan), socioeconomic disadvantage (reference = IRSD Q5 (least disadvantaged)), breast cancer stage (reference = early), number of medical services accessed during the period analysed, and death during the time period being modelled

*p value < 0.05, **p value < 0.01, ***p value < 0.001

Finally, we examined the mean co-payment per patient for each 6-month time period for PBS services, adjusting for indigenous status, remoteness, socioeconomic status, age group at diagnosis, stage of disease at diagnosis, number of PBS services during the period analysed, and death during the time period being analysed (Table 6). Co-payments were significantly lower for Indigenous women during each of the 6-month periods analysed compared with those for non-Indigenous women (ranging from 41% less during months 7–12, to 30% less during 19–24 months). There were no significant differences by remoteness in any of the 6-month periods analysed. Compared with women living in the least disadvantaged quintile (Q5), patient co-payments reduced with increasing disadvantage in the first 6 months post-diagnosis (Q1, 21% fewer; Q4, 13% fewer). Women from the most disadvantaged quintile also had 18% fewer costs during 13–18 months, 15% fewer costs during 19–24 months, 19% fewer costs during 25–30 months, and 16% fewer costs during 31–36 months.

**Table 6:** Parameter estimates of independent variables in generalized linear regression model of the co-payments for PBS prescriptions for women diagnosed with breast cancer between 1 July 2011 and 30 June 2012, Australia (weighted data presented)

	0–6 months		7–12 months		13–18 months		19–24 months		25–30 months		31–36 months	
	Ratio	Coefficient (SE)	Ratio	Coefficient (SE)	Ratio	Coefficient (SE)	Ratio	Coefficient (SE)	Ratio	Coefficient (SE)	Ratio	Coefficient (SE)
Intercept		3.9211 (0.0765)***		2.3079 (0.0779)***		2.0292 (0.0844)***		1.7807 (0.0829)***		1.3308 (0.0802)***		0.9763 (0.0892)***
Indigenous women	0.61	− 0.4926 (0.1191)***	0.59	− 0.5234 (0.1125)***	0.61	− 0.4973 (0.1206)***	0.70	− 0.3632 (0.1345)**	0.63	− 0.4557 (0.1299)***	0.68	− 0.3855 (0.1419)**
Regional	1.01	0.0059 (0.0347)	0.96	− 0.0382 (0.0338)	1.02	0.0174 (0.0356)	1.01	0.0105 (0.0354)	1.08	0.0742 (0.0364)	1.07	0.0648 (0.0386)
Remote	1.09	0.0816 (0.0583)	0.99	− 0.0081 (0.0574)	1.10	0.0935 (0.0609)	1.06	0.0536 (0.0603)	0.99	− 0.0088 (0.0619)	1.09	0.0826 (0.0658)
IRSD Q1	0.79	− 0.2370 (0.0672)***	0.91	− 0.0971 (0.0654)	0.82	− 0.1955 (0.0681)**	0.85	− 0.1580 (0.0674)*	0.81	− 0.2156 (0.0701)**	0.84	− 0.1776 (0.0735)*
IRSD Q2	0.75	− 0.2850 (0.0742)***	0.83	− 0.1845 (0.0732)*	0.84	− 0.1718 (0.0762)*	0.87	− 0.1394 (0.0756)	0.78	− 0.2486 (0.0770)**	0.83	− 0.1923 (0.0819)*
IRSD Q3	0.83	− 0.1870 (0.0479)***	0.93	− 0.0748 (0.0468)	0.92	− 0.0798 (0.0492)	0.96	− 0.0459 (0.0492)	0.90	− 0.1085 (0.0505)*	0.95	− 0.0533 (0.0535)
IRSD Q4	0.87	− 0.1405 (0.0372)***	0.95	− 0.0527 (0.0361)	0.93	− 0.0707 (0.0381)	0.96	− 0.0388 (0.0380)	0.90	− 0.1102 (0.0389)**	0.96	− 0.0422 (0.0414)
Age 45–64 years	0.87	− 0.1406 (0.0456)**	1.19	0.1725 (0.0446)***	1.08	0.0784 (0.0477)	1.11	0.1065 (0.0481)*	1.18	0.1633 (0.0498)***	1.18	0.1631 (0.0529)**
Age ≥ 65 years	0.37	− 0.9955 (0.0477)***	0.70	− 0.3612 (0.0487)***	0.66	− 0.4123 (0.0518)***	0.68	− 0.3875 (0.0525)***	0.77	− 0.2604 (0.0539)***	0.80	− 0.2206 (0.0572)***
Advanced stage	1.35	0.2996 (0.0309)***	1.03	0.0253 (0.0288)	1.02	0.0214 (0.0300)	1.04	0.0417 (0.0299)	1.08	0.0746 (0.0308)*	1.05	0.0468 (0.0323)
Unknown stage	1.09	0.0821 (0.0589)	1.07	0.0685 (0.0583)	1.13	0.1234 (0.0613)*	1.21	0.1887 (0.0620)**	1.17	0.1556 (0.0667)*	1.51	0.4111 (0.0735)***
Number of PBS services during period analysed	1.03	0.0251 (0.0010)***	1.02	0.0207 (0.0011)***	1.02	0.0179 (0.0011)***	1.02	0.0186 (0.0011)***	1.02	0.0181 (0.0011)***	1.02	0.0173 (0.0012)***
Death during period analysed	0.74	− 0.3063 (0.0536)***	1.07	0.0696 (0.0574)	0.96	− 0.0411 (0.0650)	0.85	− 0.1670 (0.0630)**	0.95	− 0.0537 (0.0607)	1.07	0.0719 (0.0648)

The ratios presented are relative to the reference group: Indigenous status (reference = non-Indigenous women), age group (reference = 18–44 years), remoteness (reference = metropolitan), socioeconomic disadvantage (reference = IRSD Q5 (least disadvantaged)), breast cancer stage (reference = early), number of PBS services accessed during the period analysed, and death during the time period being modelled

*p value < 0.05, **p value < 0.01, ***p value < 0.001

## Discussion

The total patient co-payments for the first 3 years for women diagnosed with breast cancer was approximately $21.7 million for MBS services and $14.2 million for PBS prescriptions. The average patient co-payment for MBS services during the first 3 years was $1440, with some women paying a maximum of $32,249. In addition, the average co-payments paid per patient for PBS prescriptions during the first 3 years post-diagnosis was $974, with a maximum of $5390.

We presented the costs for patient co-payments for MBS services and PBS prescriptions. A recent Queensland study estimated the median patient co-payments for all services and prescriptions billed through Medicare during the first 2 years post-diagnosis was $4192 [3]. These results are also comparable to other reports of high OOP costs in Australia following a breast cancer diagnosis [2, 4]. Both of these other studies include direct and indirect costs following a breast cancer diagnosis. Our data set did not include costs which did not incur a rebate paid by Medicare. However, our study is unique in that it describes the distribution of patient co-payments by Indigenous status, remoteness, and socioeconomic status.

We found that Indigenous women and women living in areas of socioeconomic disadvantage had significantly lower patient co-payments for MBS services during the 12 months following diagnosis, even after adjusting for the number of services used during this time. These findings may indicate that the policies in place to protect individuals and family groups from spending a high amount on patient co-payments during the year are working. These policies include lower payments for eligible Concession Card holders, as well as the Medicare and Extended Medicare Safety Nets. Once an individual or family group patient reaches the threshold set for that calendar year, then they may receive a greater proportion of Medicare rebate for out-of-hospital services [9].

In Australia, the rebate paid by Medicare is set in the Schedule (a listing of all Medicare services subsidized by the Government); however, health practitioners are able to set the fee charged for the service provided, resulting in unregulated patient co-payments. Some MBS service providers may also choose to bulk-bill patients, resulting in no patient co-payment. Some un-referred services may be eligible for bulk-bill incentives from the government [20]. In 2015, OOP expenditure in Australia was 20%, which was equal to the Organisation for Economic Cooperation and Development average (20%), but higher than the average paid in the UK (15%), New Zealand (13%), and Canada (15%) [21]. This is of concern, as it is known that people may delay or forgo healthcare due to costs [5, 7]. Previous work by some of the authors found that 21% of Australian adults with cancer skipped care due to the costs [5]. In a survey of people with cancer, 10.9% indicated that the cost of treatment influenced their decision about cancer treatment [7]. A recent study using CancerCostMod identified that on average, Indigenous Australians with cancer had lower patient co-payments for MBS services and PBS prescriptions combined compared with non-Indigenous Australians with cancer. There were also differences in the number and type of MBS services accessed between Indigenous Australians and non-Indigenous Australians [22]. Future studies should identify if there are differences in the type and number of MBS services and PBS prescriptions for women diagnosed with breast cancer.

In relation to PBS prescriptions, we found that after adjusting for the number of prescriptions, patient co-payments were significantly lower in Indigenous women and in women living in areas of socioeconomic disadvantage (Q1–4). Again, these findings may indicate that the prescriptions dispensed under the CTG scheme for Indigenous Australians, lower co-payments for people with eligible Concession Cards, and the PBS Safety Net may be protecting individuals and family groups from paying excessive patient co-payments for their prescriptions. In contrast to unregulated patient co-payments for MBS services, patients will pay up to the patient co-payment for approved PBS medications (2018 general patient, $39.50; and concession card holder $6.40) [23]. However, previous studies have found that cost is a barrier in obtaining the prescription [6, 24]. In a survey of people with cancer, 11% indicated that medications prescribed for their cancer treatment caused financial burden. Those who had a reduced income following their diagnosis reported greater financial burden due to prescribed cancer medications. Almost 12% of participants indicated that they used an alternative (over-the-counter, medication already at home, medicines from someone else) to their prescribed cancer-related medications [6].

Our study found no consistent difference in the patient co-payments paid for MBS services or PBS prescriptions by remoteness. In comparison, previous studies have reported higher patient OOP expenditure for people living outside of urban areas. A recent Western Australia study reported that of people diagnosed with one of the four most common cancers, total OOP expenditure was higher in participants residing outside of the South West region, who had private health insurance and were under the age of 65 years. This study included direct and indirect costs. The categories which accounted for the greatest proportion of expenditure were surgery, tests, accommodation, and fuel [25]. These results were similar to a Queensland study, which reported travel expenses accounting for the greatest proportion (71%) of total costs for cancer patients [12]. OOP costs were greatest for people living more than 100 km from the hospital in which they received care, compared with those who lived within 100 km from this hospital [12]. Our study was unable to estimate indirect costs, as these were not covered by Medicare.

This study has several strengths, primarily due to the use of population-based linked administrative data. We included the patient co-payment costs of all MBS services and PBS prescriptions from date of diagnosis to 36 months post-diagnosis for women diagnosed with breast cancer. The data was weighted to be representative of the Australian population. We have previously calculated that the age-standardized incidence rate of women diagnosed with breast cancer for CancerCostMod was 120.56 per 100,000, compared with the national age-standardized incidence rate of 120.42 per 100,000 for women diagnosed with breast cancer in 2012 [13]. Administrative data also overcomes potential measurement bias (poor recall, self-report, interviewer, etc.). However, administrative data also has several weaknesses. For example, the QCR does not routinely collect stage of disease at diagnosis, or breast cancer type, or socioeconomic status of individuals. Therefore, we identified the stage as ‘early’, ‘advanced’, and ‘unknown’ and used aggregated area-level data to identify socioeconomic disadvantage and remoteness. We were unable to estimate patient OOP costs which were not covered by Medicare, such as some medical services, over-the-counter medications, private prescriptions, private health insurance (including premiums and excess), travel, accommodation, food, or indirect costs due to changes in labour force participation for the patient (and their caregiver/s). These indirect costs are known to account for a high proportion of the costs to the patient [2, 12, 25]. Patient comorbidities were also excluded from the dataset and, therefore, not adjusted for in the analysis.

The sample of women diagnosed with breast cancer that was used in this manuscript was obtained from a larger dataset, CancerCostMod. The original data included had indigenous status recorded for 87% of the records [13]. In the development of this dataset, Indigenous status was imputed for records with missing or unknown Indigenous status. Missing Indigenous status is a common data limitation in Australian health studies [26]. Previously, Australian national cancer statistics have included data from five (of eight) jurisdictions only, as these jurisdictions are considered to have sufficient completeness of Indigenous status for reporting [1]. It is possible that the national statistics underestimate the true incidence of cancer in Indigenous Australians [27]; it is also possible that we have overestimated the number of new cases of cancer for Indigenous Australians.

## Conclusion

This study supports previous findings of high OOP co-payments following a breast cancer diagnosis. We also found significant differences in the patient co-payments for women diagnosed with breast cancer in Australia by Indigenous status and socioeconomic disadvantage. Although it may be difficult to predict all of the patient co-payments throughout their cancer journey, there is a call for greater transparency for patient co-payments. As costs are a potential barrier to accessing treatment, health professionals should be aware of potential co-payments which may be incurred and discuss these with the patient throughout their cancer journey.
